# Novel endoscopic delivery modality of infrared coagulation therapy for internal hemorrhoids

**DOI:** 10.1007/s00464-012-2325-1

**Published:** 2012-05-31

**Authors:** Elisabeth C. McLemore, Rudra Rai, Junaid Siddiqui, P. Patrick Basu, Mousab Tabbaa, Michael S. Epstein

**Affiliations:** 1 Department of Surgery, University of California, San Diego, San Diego, CA USA; 2 UC San Diego Medical Center, Moores Cancer Center, 3855 Health Sciences Dr., #0987, La Jolla, San Diego, CA 92093-0987 USA; 3 Johns Hopkins School of Medicine, Baltimore, MD USA; 4 Round Rock, TX USA; 5 Forest Hills, NY USA; 6 Westlake, OH USA; 7 Annapolis, MD USA

**Keywords:** Infrared coagulation, Hemorrhoids, Rectal bleeding, Colonoscopy, Endoscopy

## Abstract

**Background:**

A novel endoscopic delivery system for infrared coagulation therapy (IRC) has been designed recently. IRC is a well-established treatment for symptomatic internal hemorrhoids. Patients frequently undergo lower endoscopy before hemorrhoid treatment to eliminate other sources of bleeding. Current treatment options are difficult to perform without an anal retractor, adequate lighting, and specialized instruments. Endoscopic IRC is an attractive alternative to standard IRC, because it can be performed during the lower endoscopy.

**Technique:**

Endoscopic IRC utilizes infrared radiation generated by a control box, which is applied to the tissue through a flexible, fiber optic light guide (Precision Endoscopic Infrared Coagulator™). The light guide is placed through the colonoscope or flexible sigmoidoscope in the same chamber as other endoscopic instruments.

**Methods:**

A retrospective review was performed using a prospectively collected database. A standardized protocol was utilized in all patients. Patients graded their symptoms before and after therapy by using the visual analog symptom severity scoring system (range, 0–10). These results were analyzed by using the nonparametric Wilcoxon signed-rank test. Exact *P* values were computed by using the R function wilcox.exact.

**Results:**

A total of 55 patients underwent endoscopic IRC for predominately grade II and grade III symptomatic internal hemorrhoids (71 %). There were 22 (40 %) female patients. Posttherapy results indicated a significant improvement in global symptoms (pretreatment average global score = 2.24 vs. posttreatment average global score = 0.28; *P* < 0.0001). There have been no adverse events reported to date.

**Conclusions:**

Endoscopic IRC provides improved visibility and efficiency, allowing simultaneous treatment of symptomatic internal hemorrhoids at the time of lower endoscopy. Patients experienced significant improvement in their symptoms after a single session of endoscopic IRC. There are a variety of additional endoscopic IRC therapeutic utilities: endoscopic management of angiodysplasia, inflammation, hemostasis, and NOTES applications.

Traditional surgical hemorrhoidectomy excises both the internal and external hemorrhoid cushions and therefore is associated with a great deal of pain during the postoperative recovery period [1]. The significant pain associated with this operation has led to a variety of nonsurgical treatment options, which have been developed to treat patients with symptoms primarily related to internal hemorrhoid disease. These nonsurgical treatment options include rubber band ligation, infrared coagulation, bipolar diathermy, direct-current electrotherapy, sclerotherapy, and cryotherapy. All of these nonsurgical options cause fibrosis, scarring, shrinkage, and fixation of the internal hemorrhoid cushions and minimize symptoms of internal hemorrhoid disease [2–4].

Patients with hemorrhoid disease are frequently encouraged to undergo a colonoscopy before the initiation of treatment for hemorrhoid disease to eliminate potential neoplastic sources within the colon and rectum as a source of the patient’s symptoms [5]. Currently, the nonsurgical treatment options of internal hemorrhoid disease cannot be done without the use of an anal retractor (anoscope), dedicated lighting source, and specialized instruments necessary for the nonsurgical treatment. In addition, nonsurgical treatment options are frequently performed at a different time and location than the colonoscopy. This leads to inefficiency for both time and expense for the endoscopist, the endoscopy facility, and the patient.

Recently, an endoscopic infrared coagulator has been developed which enables the endoscopist to treat the internal hemorrhoid disease with infrared coagulation at the same time as the lower endoscopy. The Precision Endoscopic Infrared Coagulator™ is FDA-approved for the treatment of internal hemorrhoids grades I, II, and III (Table 1). The technique of infrared coagulation utilizes infrared radiation of 1–5 S pulse of energy applied onto the internal hemorrhoid tissue through a light guide. The infrared coagulator pulse of energy coagulates tissue protein and evaporates water from cells leading to coagulation, obliteration, and eventual scaring and fixation of the redundant hemorrhoid tissue.

**Table 1:** Banov internal hemorrhoid grading system [6]

Grade	Description
I	Bleeding, no prolapsed
II	Prolapse with spontaneous reduction, ±bleeding
III	Prolapse that requires manual reduction, ±bleeding
IV	Prolapse that cannot be reduced, usually both internal and external hemorrhoid components confluent from external hemorrhoid engorgement or skin tag to inner anal canal, ±bleeding

Infrared coagulation (IRC) is a well-established treatment option for symptomatic internal hemorrhoids. The current standard infrared coagulator utilizes infrared radiation generated by a tungsten-halogen lamp in a portable control box, which is applied to the hemorrhoid tissue through a solid quartz light guide at the tip of the infrared coagulator (Infrared coagulator IRC2100™, Redfield Corporation, Rochelle Park, NJ). The endoscopic infrared coagulator utilizes infrared radiation generated by a portable control box, which is applied to the hemorrhoid tissue through a flexible, fiber optic light guide (Precision Endoscopic Infrared Coagulator™, Precision Endoscopic Technologies, Annapolis, MD). The light guide is placed through the colonoscope or flexible sigmoidoscope in the same accessory chamber as other endoscopic instruments (biopsy forceps, snare cautery, endocatch bags, etc.). The endoscopic infrared coagulator allows for direct endoscopic visualization of the procedure. The improved visibility with the endoscopic application compared with the anal retractor application of infrared coagulation is an attractive feature of the new medical device.

## Methods

### Study design

A retrospective review was performed by using a prospectively collected database registry. Patient selection included age 18–80 years, rectal bleeding attributed to internal hemorrhoids on at least a weekly basis for 3 months or more, and no previous infrared therapy for internal hemorrhoids. Using a standardized protocol, each patient then underwent a thorough history and physical examination. Anoscopy, sigmoidoscopy, or colonoscopy also was performed to eliminate other sources of bleeding. Demographic data were collected exclusive of any patient-identifying information to ensure the confidentiality of the data collection process. Information pertaining to the hemorrhoid location, grade, and patient symptoms also was collected.

The patient’s hemorrhoids were graded by using the Banov scale of grade I–IV (Table 1) [6]. Patients were excluded from participation if they had a history of cancer not in remission for 5 years or more, prior hemorrhoid treatment in past 12 months, history of pelvic radiation, personal history of inflammatory bowel disease, anticoagulation therapy, concurrent anal fistula or fissure, advanced renal, cardiac disease, pulmonary disease, artificial mechanical heart valve or shunt, and inability to tolerate endoscopy with sedation. The patient’s performed self-symptom severity assessments before and after therapy. The posttherapy symptom severity evaluation was performed 6 weeks after the initial treatment. Self-symptom severity assessment included a six-point visual analog scale rating the severity of bleeding, prolapse, pain, itching, burning, and soiling on a scale of 0–10. A global score was constructed by averaging these six components.

The treatment of internal hemorrhoids with the endoscopic infrared coagulator (Precision Endoscopic Infrared Coagulator™) was performed either in an office setting or outpatient surgical setting. The treatment was performed in conjunction with a flexible sigmoidoscopy or colonoscopy. Subjects had the option to receive sedation during the procedure at their request or at the discretion of the treating physician. The treatment was performed in accordance with the methodology, and procedure sections of a standardized protocol were provided to each participating physician. The six participating physicians included specialists in gastroenterology, general surgery, and colorectal surgery experienced in diagnostic and therapeutic endoscopy, standard infrared coagulation therapy, and endoscopic and anoscopic-guided rubber band ligation. Collection, analysis, and reporting of data from the various participating physician sites were coordinated by Investigative Clinical Research, Annapolis, Maryland. The results were analyzed for statistical significance in each symptom using the nonparametric Wilcoxon signed-rank test. Exact *P* values were computed by using the R function wilcox.exact by D.O. Scharfstein, professor of biostatistics, ScD (Johns Hopkins Bloomberg School of Public Health, Baltimore, MD) [7].

### Emerging technology device

The PRECISION™ endoscopic infrared coagulator device consists of two units:

1. A control unit that houses a source of infrared energy and control circuitry, which is plugged into a standard AC electrical outlet, and
2. A single-use disposable MAXi-guide™ flexible fiber optic light guide whose proximal end is connected to the control unit by a quick-connect threaded handle, and whose distal tip is inserted into the accessory channel of a colonoscope, flexible sigmoidoscope, or other flexible endoscope.

The current configuration of the single-use disposable flexible light guide is 3.2 mm in outer diameter, and the overall length of this flexible light guide is 300 cm. When in use, visible light and infrared energy enter the proximal end of the fiber optic and are passed through its entire length to the distal tip. As the distal tip is placed in contact with tissue, infrared energy is transferred to the tissue, rapidly increasing the temperature and coagulating the tissue. The PRECISION™ endoscopic infrared coagulator device was cleared to market in the United States by the Food and Drug Administration in June 2009 through the 510(k) procedure (K083275). This device also carries the CE Mark (CE 542048) and US Patent 7,977,658.

### Description of the procedure

After explanation of the procedure and obtaining informed consent, the patients were placed in the left-lateral decubitus position. Visual and digital inspection of the anus and rectum were performed, and any abnormal findings were noted. The patient’s had the option to receive sedation at his or her request. Either flexible colonoscopy or sigmoidoscopy was performed with an instrument having at least a 3.7 mm instrument channel. After evaluation of the bowel, the instrument was retroflexed, and the anorectal junction was inspected carefully. The internal hemorrhoid area was evaluated, and any bleeding, prolapse, or other abnormal findings were noted.

**Fig. 1:**
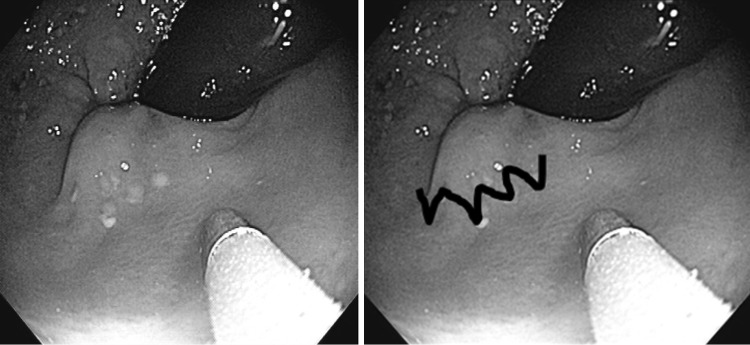
Overlapping “W”: semi-lunar spot endoscopic IRC technique. *IRC* infrared coagulation

The endoscope was straightened, and the PRECISION™ Endoscopic Infrared Coagulator single-use flexible fiber optic probe inserted into the endoscope through the biopsy cap. Before insertion, it was gently lubricated on its entire length. The flexible fiber optic probe was inserted using gentle short strokes, taking care not to kink the fiber optic cables upon insertion. The fiber optic probe was advanced until it protruded 1–2 cm from the tip of the scope. The endoscope was then retroflexed in the rectum, and the hemorrhoid columns were identified. Endoscopic infrared coagulation treatment was applied by gently opposing the tip of the probe to the tissue just above the engorged internal hemorrhoid tissue column. The probe was held in place for up to 3–5 S. The probe was then moved to an adjacent location in an overlapping semilunar “W” fashion (Fig. 1). Treatment was performed on one to three internal hemorrhoid quadrants at the discretion of the endoscopist.

### Follow-up

Follow-up evaluation was performed at approximately 6 weeks by anoscopy or consultation. A second session was allowed and performed approximately 90 days after the initial therapy if residual symptoms or tissue required additional therapy. The patients were given instructions to maintain soft stools for several days after therapy with stool softeners and/or appropriate fiber intake.

## Results

Fifty-five patients underwent endoscopic infrared coagulation therapy using the PRECISION™ Endoscopic Infrared Coagulator. The grade of internal hemorrhoids was predominately grade II and III (Table 2). There were 22 (40 %) female patients (Table 3). Pretreatment bowel habits and symptoms associated with internal hemorrhoids are displayed in Table 4. No intention-to-treat analysis was performed, because only three patients were excluded. Two patients developed psychiatric disturbances that led to an inaccurate reporting of symptoms. The third patient was excluded due to a diagnosis of hepatitis C and cirrhosis after initial evaluation.

**Table 2:** Internal hemorrhoids grade and location

Internal hemorrhoid data					
	Grade I	Grade II	Grade III	Grade IV	Not recorded
Internal hemorrhoids (# patients)	6	19	20	3	7

	Left lateral	Right anterior	Right posterior	Not recorded
Location of hemorrhoids (# incidence)	33	22	19	18

**Table 3:** Patient demographics

No. of patients	55
Median age (year)	46 (16–77)
Female	40 % (22)
History of childbirth	32 % (18)
History of episiotomy	16 % (9)
Family history colorectal cancer	16 % (9)
Prior colonoscopy	78 % (43)

**Table 4:** Bowel habits and hemorrhoid symptoms

No. of years hemorrhoids present	0–10 years	>10 years	Unknown
	62 % (34)	22 % (12)	16 % (9)
Blood in stool	Frequently	Occasionally	Never
	24 % (13)	58 % (32)	18 % (10)
Hemorrhoids protrude	31 % (17)	29 % (16)	34 % (19)
Straining with BM	29 % (16)	49 % (27)	22 % (12)
Constipation	18 % (10)	60 % (33)	22 % (12)
Hard and firm stools	30 % (17)	56 % (31)	12 % (7)
Feel lump in rectum	18 % (10)	24 % (13)	55 % (30)
Pain with BM	18 % (10)	30 % (17)	51 % (28)
Drainage around rectum	3 % (2)	24 % (13)	73 % (40)
Soil underwear	5 % (3)	27 % (15)	65 % (36)
Laxative use	7 % (4)	29 % (16)	68 % (34)
Fiber supplement use	22 % (12)	34 % (19)	38 % (21)

*BM* bowel movement

Posttherapy results indicated an average improvement of 87.6 % in global symptoms attributed to internal hemorrhoids (pretreatment average global score = 2.24 vs. posttreatment average global score = 0.28; *P* < 0.0001; Table 5). Fifty-three patients received a single treatment, and two patients underwent a second treatment session. In 49 patients (89 %), the posttreatment severity scores were the same or lower than the pretreatment severity scores on all six components. There were six patients with an increase in at least one component. In these six patients, the overall global scores all decreased; one patient experienced an increase in bleeding (from 1.2 to 2.2); two patients experienced an increase in prolapse (0.0–3.3 and 0.0–2.0), one patient experienced an increase in pain (0.0–0.7), two patients experienced an increase in itching (0.0–2.9 and 1.7–1.9), none experienced an increase in burning, and one patient experienced an increase in soiling (0.0–0.7). There have been no adverse events reported to date.

**Table 5:** Average pre- and posttreatment symptom scores

Symptom	Pretreatment average (median) score	Posttreatment average (median) score	*P* value*
Bleeding	3.81 (3.1)	0.37 (0)	<0.0001
Prolapse	1.47 (0)	0.39 (0)	0.0005
Pain	2.37 (1.9)	0.16 (0)	<0.0001
Itching	2.29 (1.5)	0.36 (0)	<0.0001
Burning	2.33 (1.5)	0.14 (0)	<0.0001
Soiling	1.16 (0)	0.23 (0)	0.0001

* Exact *P* values computed using the R function wilcox.exact

## Discussion

Symptomatic internal hemorrhoid engorgement is a common human condition. Symptoms may include bleeding, itching, burning, tissue prolapse, and fecal seepage. Internal hemorrhoids that are unresponsive to dietary modification and topical and suppository agents may be treated with rubber band ligation, coagulation therapy (infrared beam, electric current, CO_2_ laser, ultrasonic energy, cryotherapy), or sclerosing agents. These treatment options require the use of an anal retractor, adequate lighting, and specialized instruments. These requirements often restrict the utilization of these treatment options at the time of screening colonoscopy for rectal bleeding. These limitations delay treatment of the patient’s symptoms. In addition, this is an inefficient use of time and resources for the patient, physician, and potentially the health care system.

The endoscopic infrared coagulator is a novel and attractive alternative to standard infrared coagulation, because it can be performed at the same time as the lower endoscopy through the accessory channel of the endoscope. Endoscopic delivery of infrared coagulation offers improved lighting and visualization compared with current anoscopic guided therapy for symptomatic internal hemorrhoids. After a single session of endoscopic infrared coagulation, patients in this study experienced a statistically significant improvement of their symptoms related to internal hemorrhoids.

The authors readily acknowledge that there are several limitations to this preliminary study. This study utilizes the patients as their own internal controls by comparing patient self-scoring of the severity of symptoms attributed to hemorrhoid disease before and after therapy. The study did not utilize ultrasound to guide the site of infrared coagulation application or monitor reduction in venous blood flow after the procedure. Finally, the study did not compare the efficacy of the new technology with the current standard of care delivery mode for infrared coagulation therapy for internal hemorrhoids.

Despite these limitations, the improved visibility of the Precision Endoscopic Infrared Coagulator™ without the need for anal retractors and specialized light source is an attractive feature for the treatment of symptomatic internal hemorrhoids. The use of the endoscopic infrared coagulation allows for simultaneous diagnostic and therapeutic endoscopy for rectal bleeding. If symptoms persist or recur, additional treatments can be performed with endoscopic infrared coagulation therapy or other nonoperative techniques as previously described at the discretion of the physician and patient.

Future clinical trials are being developed to evaluate further the effectiveness of the device for obtaining hemostasis in the setting of angiodysplasia, radiation proctitis, and endoscopic biopsy/polypectomy. Endoscopic ablation of esophageal, anal, and rectal dysplasia is another field of potential therapeutic utility. In addition, a variety of applications are undergoing investigation in the rapidly evolving arena of natural orifice transluminal endoscopic surgery (NOTES).
